# Repositioning of Tamoxifen in Surface-Modified Nanocapsules as a Promising Oral Treatment for Visceral Leishmaniasis

**DOI:** 10.3390/pharmaceutics13071061

**Published:** 2021-07-10

**Authors:** Débora Faria Silva, Levi Eduardo Soares Reis, Marina Guimarães Carvalho Machado, Douglas Daniel Dophine, Vinicius Roberto de Andrade, Wanderson Geraldo de Lima, Margareth Spangler Andrade, José Mário Carneiro Vilela, Alexandre Barbosa Reis, Gwenaelle Pound-Lana, Simone Aparecida Rezende, Vanessa Carla Furtado Mosqueira

**Affiliations:** 1 Laboratory of Clinical Research, Pharmacy School, Federal University of Ouro Preto, Ouro Preto 35400-000, Brazil; debora.faria@ufop.edu.br (D.F.S.); douglasdophine@gmail.com (D.D.D.); vinicius.roberto@aluno.ufop.edu.br (V.R.d.A.); rezende@ufop.edu.br (S.A.R.); 2 Laboratory of Pharmaceutics and Nanotechnology, Pharmacy School, Federal University of Ouro Preto, Ouro Preto 35400-000, Brazil; marinaguimacar@gmail.com (M.G.C.M.); gwenicioui@gmail.com (G.P.-L.); 3 Laboratory of Immunopathology, Post-Graduate Program in Biological Sciences, Federal University of Ouro Preto, Ouro Preto 35400-000, Brazil; levieduardo@yahoo.com.br (L.E.S.R.); alexreis@ufop.edu.br (A.B.R.); 4 Laboratory of Morphopathology, Institute of Exact and Biological Sciences, Federal University of Ouro Preto, Ouro Preto 35400-000, Brazil; wanderson@ufop.edu.br; 5 Center–SENAI/CETEC, Laboratory of Nanoscopy, Belo Horizonte 31035-536, Brazil; margarethcasa@gmail.com (M.S.A.); jvilela@fiemg.com.br (J.M.C.V.)

**Keywords:** tamoxifen, repurposing, leishmaniasis, efficacy, nanocapsule, physicochemical characterization, PEG-PLA, drug release

## Abstract

Standards of care for human visceral leishmaniasis (VL) are based on drugs used parenterally, and oral treatment options are urgently needed. In the present study, a repurposing strategy was used associating tamoxifen (TMX) with polyethylene glycol-*block*-polylactide nanocapsules (NC) and its anti-leishmanial efficacy was reported in vivo. Stable surface modified-NC (5 mg/mL of TMX) exhibited 200 nm in size, +42 mV of zeta potential, and 98% encapsulation efficiency. Atomic force microscopy evidenced core-shell-NC. Treatment with TMX-NC reduced parasite-DNA quantified in liver and spleen compared to free-TMX; and provided a similar reduction of parasite burden compared with meglumine antimoniate in mice and hamster models. Image-guided biodistribution showed accumulation of NC in liver and spleen after 30 min post-administration. TMX-NC reduced the number of liver granulomas and restored the aspect of capsules and trabeculae in the spleen of infected animals. TMX-NC was tested for the first time against VL models, indicating a promising formulation for oral treatment.

## 1. Introduction

According to the World Health Organization, most cases of visceral leishmaniasis (VL) occur in India, East Africa, and Brazil, where the main etiologic agent is *Leishmania (Leishmania) infantum,* transmitted between definitive hosts by insects of the subfamily *Phlebotominae*. Visceral Leishmaniasis is an infectious parasitic disease that is lethal in over 95% of the untreated cases [1].

Chemoterapy of VL currently relies on pentavalent antimonial compounds, which present high toxicity and are becoming less effective due to the emergence of resistant parasites [2]. Furthermore, treatment requires daily intramuscular (IM) or intravenous (IV) injections for at least 20 days, which causes poor patient adherence to treatment [3]. Therefore, new drugs and new formulations allowing the use of the oral route of administration are in pressing need.

The strategy of drug repurposing has been used as an alternative for new treatment options with reduced time of development for different neglected diseases [4,5,6]. In this context, the triphenylethylene class of molecules represents a privileged chemical scaffold showing activity against parasites, virus, bacteria, and fungi [7]. Among them, tamoxifen (TMX) is active against *L. braziliensis*, *L. major*, *L. infantum,* and *L. donovani* and shows good in vitro activity against promastigotes and amastigotes of *L. infantum* (IC_50_ = 2.4 ± 0.3 µM) [8]. Furthermore, TMX administered over 15 days by the intraperitoneal (IP) route has anti-leishmanial efficacy in vivo in *L. infantum*-infected hamsters [8]. Syrian hamster is a better animal model to evaluate the efficacy of experimental infection with *Leishmania spp.* because, unlike mice, hamsters exhibit no spontaneous cure, and therefore more closely simulates the course of human infection [9,10]. TMX is a drug used in the treatment of breast cancer since 1971, and it acts as a selective estrogen receptor modulator [11]. However, TMX action against *Leishmania spp.* is independent on binding to the estrogen receptor. TMX induces alkalinization of the phagolysosome within macrophages [8] and it interferes strongly in *Leishmania* sphingolipid biosynthesis, causing parasite death [12]. Recently, it was shown that the effect of TMX on *Leishmania* is mediated, at least in part, by disorders in the parasite’s membrane, sufficient to trigger a series of lethal events [13]. Furthermore, TMX has the ability to inhibit protein kinase C, which appears to play a role in its biological activity [14].

The oral route is considered the more affordable, comfortable, and easiest route of drug administration from a physiological perspective, with the best patient compliance for the treatment of the majority of the diseases, as well as in leishmaniasis. However, to date, no effective oral formulation is commercially available to treat leishmaniasis. Though already used by oral route, TMX has some limitations due to its low water solubility (2.9 ± 1.0 nM at 25 °C), low solvation, high lipophilicity (log *P* = 7.1) and high pKa 8.5 [15,16]. TMX converted to its citrate salt to increase solubility in water in commercial tablets [17] with an estimated bioavailability of around 94% in humans [18]. Furthermore, the oral route is the best choice, because TMX suffers first-pass metabolism and results in high systemic availability of its main metabolite, which is active in breast cancer treatment and most likely also against *Leishmania* [19]. However, the effects of the two major TMX metabolites on *Leishmania spp*. remain undetermined [20]. An important aspect of TMX repositioning in the treatment of leishmaniasis was recently reported by Coelho et al. (2015) [21], who used several strategies to generate resistance in *L. amazonensis*. The parasites obtained developed resistance to miltefosine, but there were no indications of resistance to TMX, which suggests a mechanism of action less favorable to the development of resistance.

Nanoencapsulation is an attractive strategy to improve the efficacy and reduce the toxicity of various compounds, particularly in parasitic diseases [22,23]. Nanocarriers can improve the pharmacokinetic profile of lipophilic substances and dramatically reduce the drug toxicity, as extensively reported for amphotericin B associated with different types of nanocarriers [24]. Well-designed drug delivery systems can reduce blood clearance, modify the interactions with host cells and pathogens, and reach specific target cells and tissues in higher concentrations, increasing the therapeutic effect and/or reducing the toxicity of the encapsulated drug [25]. Polymeric nanocapsules (NC) with an oily core have been developed to increase the dispersion of lipophilic drugs in aqueous media, to protect drugs against chemical degradation, to control the release of drugs in biological fluids as well as to improve the absorption in the gastrointestinal tract with increased permeability across the intestinal epithelia [22,26,27,28]. Recently, the association of the anti-*Trypanosoma cruzi* active compound lychnopholide with polyethyleneglycol-*block*-polylactide nanocapsules (PEG-PLA NC) improved its efficacy by IV and oral route in the acute and chronic phases of the experimental *T. cruzi* infection in mice and prevented its cardiotoxicity in vivo [29,30,31,32]. Similarly, NC containing artemether proved to be a useful nanocarrier for experimental oral treatment of malaria in mice in terms of efficacy and protection from drug cardiotoxicity [33]. In line with the literature and considering the promising results previously obtained by using PEG-PLA NC administered orally against parasites of the trypanosomatidae family, the nanoencapsulation potential on the repositioning of TMX loaded in PEG-PLA NC was examined for the oral treatment of VL. This study reports the development and physicochemical characterization of TMX-NC, its release kinetics, NC biodistribution after oral administration and evaluation of TMX-NC anti-leishmanial efficacy in two animal models, mice, and hamster. Efficacy was determined by quantifying the parasite burden and by examining the histopathology of the liver and spleen of animals experimentally infected with *L. infantum*.

## 2. Materials and Methods

### 2.1. Materials

TMX citrate (≥99% purity; manufacturer batch TC/006/02/16) was obtained from Fresenius Kabi AG (Pune, India) and was converted to TMX free base as described in the Supplementary Materials. The mPEG-PLA diblock copolymer [monomethoxi-polyethylene glycol-*block*-poly(*d,l*)-lactide] (Mn 22,000 g/mol, Ð 1.26) was synthesized in our laboratory by ring opening polymerization of *D*,*L*-lactide from a PEG block of Mn 5000 g/mol, and characterized, as previously described [34]. Miglyol^®^810N (capryc/caprylic triglyceride, medium chain triglyceride) was provided by Sasol GmBH (Hamburg, Germany). Lipoid^®^S75 (soybean phospholipids with ~75% phosphatidylcholine) was provided by Lipoid GmBH (Ludwigshafen, Germany). All other reagents and compounds used are listed in detail in the Supplementary Materials.

### 2.2. Preparation of Tamoxifen Nanocapsules

The method of extraction of TMX base from TMX citrate is described in detail in the Supplementary Materials. The TMX-loaded NC were prepared by polymer deposition followed by solvent displacement method first described by Fessi et al., 1989 [35]. Briefly, Miglyol^®^810N (50 μL), Lipoid^®^S75 (15 mg), and TMX (0.5, 1.5, 2.5, 5.0, 7.5 and 10.0 mg per ml of final NC suspension) were dissolved in ethanol (2 mL); the polymer (PEG-PLA, 24 mg) was dissolved in acetone (2 mL) and the organic phases combined. The resulting solution was poured via a syringe into ultrapure water (8 mL) under moderate magnetic stirring. The resulting colloidal dispersion was kept under magnetic stirring for 10 min. The solvents were evaporated under reduced pressure at 35 °C in a rotary evaporator to a final volume of 2 mL. Blank-PEG-PLA NC (Blank-NC) were obtained similarly in the absence of TMX. All formulations were stored at 4 °C until use.

### 2.3. Physicochemical Characterization

The *Z*-average hydrodynamic diameter (*D*_h_) and population size polydispersity index (PdI) of TMX-NC formulations were determined by dynamic light scattering (DLS) on a Zetasizer NANO ZS instrument (Malvern Instruments, Malvern, UK) at a backscattering angle of 173° at 25 °C. Samples were analyzed after 1:200 dilution in 0.22 μm-filtered ultrapure water, considering values of 0.8872 cP for water viscosity and 1.330 for water refractive index. Ten measurements were recorded for each sample in three independent NC batches of the same formulation and processed using multimodal analysis, using Contin algorithm based on the Non Negative Least Squares method applied for multimodal samples. The zeta potential was determined by DLS coupled to microelectrophoresis on the same equipment with 1:200 sample dilution in 1 mM NaCl. Values reported are the means ± standard deviation of at least three different batches of each TMX-NC formulation (0.5 mg/mL to 10.0 mg/mL). Blank-NC were characterized under the same conditions. The parameters mentioned above were evaluated at the day of preparation and every 15 days during 6 months, in order to evaluate their stability upon storage at 4 °C. All studies were conducted in triplicate.

### 2.4. Atomic Force Microscopy Imaging and Analyses

Blank-NC and TMX-NC (5.0 mg/mL) were analyzed by Atomic Force Microscopy (AFM, Digital Instruments Inc., Santa Barbara, CA, USA) in a Dimension 3000 equipment monitored by a Nanoscope IIIa controller from Digital Instruments (Santa Barbara, CA, USA) using the NanoScope Analysis software 5.31rl (5.30r3sr3) for data acquisition. A sample of each formulation (5 μL) was deposited on freshly cleaved mica, spread and dried with a stream of argon. Measurements were performed in air at room temperature, in tapping mode, using a silicon probe with cylindrical tip of 228 μm radius, resonance frequencies of 75–98 kHz, spring constants of 3.0–7.1 N/m, and a nominal tip curvature radius of 5 nm. The geometric diameters of the particles were measured at half-height in topographical profiles. Images were recorded in scanning areas of 2.0 × 2.0 µm and 1.6 × 1.6 µm with a scan rate of 1 Hz.

### 2.5. Tamoxifen Encapsulation

The total TMX content in NC colloidal dispersion was determined as follows. A sample of the formulation (100 µL) was vortex-mixed for 5 min with 5.0 mL of acetonitrile to disrupt the NC and release TMX. Then, the sample was centrifuged at 500× *g* for 30 min, the supernatant was filtered (0.45 μm filter, Millipore^®^) and a 60 µL-aliquot was analyzed by High Performance Liquid Chromatography-Ultraviolet detection (HPLC-UV, Waters, Milford, MA, EUA) as described in detail in the Supplementary Materials. To determine the TMX content in the external phase of the NC colloidal dispersion, 400 µL of NC sample was placed in an ultrafiltration device (Amicon^®^, 50,000 Da cut-off, Millipore, Burlington, MA, USA) and centrifuged at 500× *g* for 30 min (Centrifuge 5415 D, Eppendorf, Hamburg, Germany). The ultrafiltrate (100 µL) collected at the bottom compartment of the device was diluted with acetonitrile (1:1) vortex-mixed (Vortex Instrument, IKA, Staufen, Germany) and TMX quantified by HPLC-UV. All analyses to determine TMX concentrations were performed in triplicate at the day of NC preparation and after 6 months. TMX percentage of loading was calculated as the difference between the total TMX in the NC suspension and the free TMX in the aqueous phase (ultrafiltrate) divided by the total TMX in the NC suspension × 100. TMX encapsulation efficiency was calculated as the difference between the total TMX in the NC suspension and the free TMX in the aqueous phase divided by the TMX weighed and used to prepare the formulation (feed). Furthermore, it was calculated that the payload, which is representative of the ratio of the mass of TMX to the total mass of NC constituents. The drug payload gives information on the capacity of the nanoparticles to transport TMX.

### 2.6. In Vitro Tamoxifen Dissolution

TMX equilibrium solubility was determined in simulated gastric fluid (SGF) without pepsin (pH 1.2) and simulated intestinal fluid (SIF) without pancreatin (pH 6.8) media, both prepared following US Pharmacopeia (USP) XXIII [36] as described in detail in the Supplementary Materials. (Table S1, supplementary materials). TMX dissolution at fixed times was determined in simulated gastric fluid (SGF) without pepsin (pH 1.2) for 2 h and followed for 2 h and 4 h in simulated intestinal fluid (SIF) without pancreatin (pH 6.8) media, to simulate in vivo conditions in individualized flow-through cell dissolution apparatus in closed system with tubes coupled to 0.22 µm syringe filters (kindly borrowed by Sotax AG-Brazil). The crushed tablets of tamoxifen citrate and 5 mg/mL of TMX-NC were properly diluted in the media to obtain final concentration of 0.1 mg/mL that correspond to sink conditions previously determined (<10% of saturation solubility). The entire system was mantained at 37 °C under continuous mechanical shaking. The amount of TMX dissolved at fixed times in the release media was determined by aditional filtration of withdraw media from system in 100,000 Da ultrafree devices (Millipore) to separate the filtrate from TMX undissolved in the NC (Table S1, supplementary materials). This dissolution experiment was conducted in triplicate. The filtrate samples were dissolved in mobile phase and kept under vortex mixing for 15 min, centrifuged at 500× *g* for 15 min, and the supernatant was filtered and assayed by HPLC-UV as described in supplementary materials. Experiments were conducted in triplicate.

### 2.7. Parasites and Cell Culture

*L. infantum* strain (MCAN/BR/2008/OP46) used in this study was first isolated from a symptomatic dog from Governador Valadares, Minas Gerais, Brazil and subsequently typed by restriction fragment length polymorphism-polymerase chain reaction (PCR-RFLP) as *L. infantum*, and denominated OP46 [37]. Parasites were cultured at 23.5 ± 1 °C in Grace’s Insect Medium supplemented with 10% FBS, 2 mM *L*-glutamine and 100 U/mL G penicillin, pH 6.5 (Grace’s 10% FBS). When the parasite culture reached the stationary phase of growth, promastigotes were collected, washed in sterile PBS (pH 7.2), and counted.

### 2.8. Experimental Animals and Ethical Statements

Female isogenic BALB/c mice and female Swiss mice, aged between 5 and 7 weeks and weighing 25–30 g, and female Syrian golden hamsters *Mesocricetus auratus (M. auratus*), aged between 6 and 8 weeks and weighing 60–80 g were obtained from the Central Animal Facility of Federal University of Ouro Preto (CCA/UFOP). The animals were randomly distributed in cages containing five animals per group and maintained at room temperatures ranging from 21 to 25 °C, under 12 h day/night cycles with water and a commercial rodent diet (Nuvilab CR-II, Sao Paulo, Brazil) ad libitum. All adopted procedures and experimental conditions used in this study were in accordance with the National Council on Animal Experiments and Control (CONCEA, Brazil) guidelines and approved by the Ethics Committee in Animal Research at UFOP under protocol numbers 2017/14 (BALB/c), 2676151019 (Swiss mice) and 2017/63 (Syrian golden hamsters).

### 2.9. Infection and Treatment Regimens

Female BALB/c mice were divided into non-infected and *L. infantum*-infected groups (*n* = 5 animals per group). Non-infected animals were treated during 15 days with PBS by IV route. *L. infantum*-infected groups were inoculated with 1 × 10^7^ promastigotes through the tail vein. Seven days post-infection, the animals received the treatment regimens during 15 days as follows: (a) sterile PBS; (b) blank-NC with excipients equivalent to 20 mg/kg/day; (c) blank-NC with the excipients equivalent to 40 mg/kg/day; (d) free-TMX: TMX suspensions dispersed in water containing 1% (wt./v) methylcellulose at 40 mg/kg/day (12 mg/mL TMX citrate solutions in 150 mM NaCl); (e) M.A. at 20 mg/kg/day; (f) TMX-NC at 20 mg/kg/day and (g) TMX-NC at 40 mg/kg/day. Except for the groups (a) and (e), all animals received treatment by oral gavage. Two weeks after the end of treatment, animals were euthanized by overdose of thiopental (50 mg/kg) and xylazine (10 mg/kg) given by the IP route. Two independent experiments were performed, the first one with *n* = 5 and the second one with *n* = 3.

Female Syrian golden hamsters were infected with 1 × 10^8^ promastigotes by IP route. Three weeks post-infection, animals (*n* = 5) were treated as described for BALB/c mice. Eight weeks after the end of treatment, animals were euthanized by overdose of ketamine (600 mg/kg) and xylazine (30 mg/kg) given by the IP route. Liver and spleen of BALB/c mice and Syrian golden hamsters were collected and weighed to determine the parasite burden and to perform histopathological analyses.

### 2.10. Parasite Quantification in Tissues

The parasite burden associated to the liver and spleen were quantified by real time quantitative-Polymerase Chain Reaction (qPCR), as previously described [38]. Total genomic DNA was extracted from approximately 20–30 mg of tissue using the Wizard TM Genomic DNA Purification Kit (Promega H, Madison, WI, USA) according to the manufacturer’s protocol. The concentration of DNA obtained from tissues was determined with a NanoDrop^®^ 2000 spectrophotometer (Thermo Scientific, Waltham, Massachusetts, EUA) and the quality of the samples was measured by the 260/280 nm ratio between 1.8 and 2.0. The DNA samples were frozen at −20 °C until further analyses. A standard curve was composed with *L. infantum* DNA (MCAN/BR/2008/OP46 strain) extracted from 1.0 × 10^8^ promastigotes/mL using the CTAB method as previously described by Moreira, et al., 2012 [38]. Briefly, each 96-well reaction plate used to determine DNA by qPCR contained a standard curve in triplicate (efficiency 96%; r^2^ = 0.99), negative control (no DNA) and control genes. A standard curve was generated with DNA extracted from a *L. infantum* cell culture quantified in a Neubauer chamber, containing 10^5^ parasites. Serial dilutions (1:10) of the extracted DNA were placed in amplification plate wells, in triplicate. The 7500 Software v.2.0.1 (Applied Biosystems, Waltham, Massachusetts, EUA) was used to determine a threshold of fluorescence intensity referred to as “Ct” and generate the standard curve relating the fluorescence intensity in the wells to the number of parasites present in the initial culture. Fluorescence measurements of serial dilutions of the extracted DNA suspensions obtained from infected tissues were used to identify the concentration corresponding to 100 ng of total DNA, from which the DNA concentration in the tissues was determined and thus the number of parasites per mg of extracted tissue. PCR was carried out in a final volume of 10 µL containing template DNA (100 ng); primers (0.3 μM); and 1× SYBER GREEN reaction master mix (Applied Biosystems, USA). The selected primer pair (forward: 5′ AGC GCC TCA CCA CGA TTG 3′; reverse: 5′ AGC GGG CAC CGA AGA GA 3′; GenBank accession number AF009147) amplified a 90 base pair (bp) fragment of a single-copy of the DNA polymerase gene of *L. infantum*. In order to verify the integrity of the samples, primers were used for murine TNF-247 α, (5′ TCCCTCTCATCAGTTCTATGGCCCA 3′; 5′CAGCA AGCATCTATGCACTTAGACCCC 3′) amplifying a 170-bp product [39]. PCR conditions were as follows: an initial denaturation step at 95 °C for 10 min followed by 40 cycles of denaturation at 95 °C for 15 s and annealing/extension at 60 °C for 1 min. All samples were run on MicroAmp^®^ Optical 96-well Reaction Plates sealed with MicroAmp^®^ Optical Adhesive Film (Applied Biosystems). The results were expressed quantitatively by the number of amastigotes/mg of tissue (spleen and liver). Two independent experiments were performed for each sample.

### 2.11. In Vivo Imaging of Biodistribution of Fluorescently Labeled Nanocapsules after Oral Administration

Labeled NC were prepared by adding IR780, a near-infrared lipophilic dye, in the organic phase before mixing with the aqueous phase, as described in the NC preparation procedure. The fluorescent NC biodistribution was analyzed semi-quantitatively using in vivo fluorescence imaging technique. IR780 was chosen as a fluorescent dye because of its high fluorescence quantum efficiency, and high lipophilicity at near infrared wavelengths, where the tissue penetration is higher [40]. The final IR780 concentration in the suspension was 27 µg/mL. Swiss mice received 200 µL of IR780-labeled NC (0.2 mg IR780/kg body weight) by oral gavage. Non-fluorescent NC and IR780 solution (dimethylacetamide:PEG 40:60 diluted 15-fold in isotonic glucose solution) were used as control groups. Fluorescence images were captured 30 min after oral administration using an in vivo animal optical imaging system (IVIS Lumina III, PerkinElmer, Waltham, Massachusetts, EUA). During the imaging procedure, isoflurane at 2.5% in oxygen was used as an inhalant anesthetic. Excitation and emission wavelengths were 740 and 790 nm, respectively. Regions of interest (ROI) were delimited using a circle of 0.9 cm in diameter to calculate the fluorescent intensity in the liver and spleen, and the same non-target area (heart) was used in calculation, because it exhibits similar blood flow.

### 2.12. Histophatological Analysis

Liver and spleen samples obtained from non-infected (healthy), infected animals and infected and treated animals were collected. The samples were fixed in 10% buffered formaldehyde, pH 7.4, for at least 48 h, processed according to routine histopathological techniques and embedded in paraffin blocks. Four-µm sections were obtained using a microtome, followed by hematoxylin-eosin (H&E staining). Images were digitalized at the Multiuser Laboratory of NUPEB/UFOP using a Leica^®^ DM5000 optical microscope through the Leica Application Suite software (version 2.4.1) and the general damages were qualitatively observed.

### 2.13. Statistical Analysis

The significant differences in physicochemical characterization and in vitro release of TMX-NC values between each experimental formulation were determined by unpaired student *t*-test. Parasite burden was first checked for normal distribution using the Kruskal-Wallis test. Normally distributed data were analyzed through one-way-ANOVA followed by Dunn’s multiple comparison post hoc test with GraphPad Prism version 6.01 (GraphPad Software, San Diego, CA, USA). The level of significance was set at *p* < 0.05 with 95% confidence interval.

## 3. Results

### 3.1. Tamoxifen PEG-PLA Nanocapsules Development

In this study, the effect of the several factors was investigated, including TMX concentration (0 to 5.0 mg/mL), ethanol/acetone ratio (1:1, 2:2, 3:1 *v*/*v*), the volumes of oil and aqueous phases and their ratios (1:2 or 1:4 *v*/*v*) on the D_h_ and PdI of TMX-NC. These variables were studied in order to obtain NC with regular size distribution and no TMX precipitation. Characterization data is shown in Table 1. All blank-NC formulations (F1, F4, F7, and F10) had Z-average D*_h_* smaller than 200 nm, independent of the oil/water phase ratio. The higher the volume of the liquid phases the lower the NC mean size. For the same ratio of phases and volumes, the higher the TMX concentration in the formulation, the higher the D_h_ and particle PdI. Increasing the phase volumes in the same proportion between the organic and aqueous phase significantly reduced the D_h_ for all TMX concentrations. An increase in the ethanol/acetone volume ratio considerably affected the TMX-loaded NC sizes, reducing the D_h_ (F5, F6, F8, F9) with no significant differences between 3:1 and 2:2 ratios. However, a 2:2 ethanol/acetone ratio led to narrower size dispersity (*p* < 0.05). The increase in aqueous phase proportion (1:4) produced a dramatic increase in D*_h_* and PdI only for TMX-loaded NC (*p* < 0.05) (F11, F12). The formulation F6 showed the lowest mean size and PdI value, while the largest amount of encapsulated TMX was achieved by using the ethanol/acetone ratio of 2:2 (*v*/*v*), 4.0 mL of organic phase and 8.0 mL of aqueous phase and the ratio 1:2 (*v*/*v*) oil phase/aqueous phase. Thus, F6 was chosen for subsequent in vitro physicochemical studies.

### 3.2. Physicochemical Characterization and Stability of Nanocapsules

**Figure 1:**
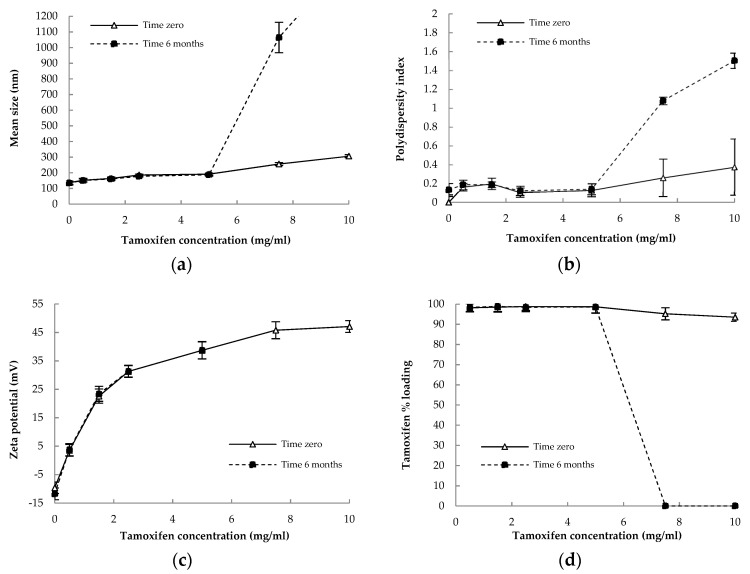
(**a**) Mean size; (**b**) Polydispersity index; (**c**) Zeta potential and (**d**) Percentage of tamoxifen loading in NC of different TMX concentration, just after preparation (time zero) and after 6 months of storage at 4 °C. Data are mean ± standard deviation of the measurements of three different NC batches.

The characterization data of NC formulations prepared with TMX concentrations from 0.0 to 10.0 mg/mL are given in Table 2. The stability of the NC as a function of time (just after preparation and after 6 months of storage) in terms of zeta potential, mean sizes, PdI, and % of loading yield as a function of TMX concentration in the NC are shown in Figure 1. The HPLC-UV method used to quantify TMX in polymeric NC was found to be selective, precise, and accurate in the range 0.5–25 µg/mL, which was suitable to determine the efficiency of encapsulation and percentage of TMX loading.

Macroscopically, TMX-NC and blank-NC presented a milky homogeneous aspect after preparation (data not shown). The Z-average D_h_ of the NC clearly increased with increasing TMX concentrations. The NC with up to 5.0 mg/mL of TMX showed narrow size distributions with D_h_ lower than 200 nm. However, above 5.0 mg/mL, the mean D_h_ were larger than 200 nm and the PdI increased significantly, indicating that colloidal stability may be compromised upon storage. The zeta potential of the NC was largely influenced by TMX, and values increased with increasing TMX concentrations. Values up to 1.5 mg/mL induced low surface charge at the NC surface (<30 mV in modulus) related to PEG steric effect at the NC surface. Zeta potential values indicate that part of the encapsulated TMX was located at the NC surface, with values ranging from −9 mV for blank-NC to +45 mV at the highest TMX concentration (10.0 mg/mL) (Figure 1). Above the concentration of 7.5 mg/mL of TMX, the NC surface was fully charged with positive ions and no additional increase in zeta potential is observed (Figure 1).

As TMX in its free base form is more lipophilic than its citrate salt, TMX base was used to improve the payload in the oil core of the NC. The TMX loading yield was very high for all TMX formulations, demonstrating a high drug affinity by the oil core of the NC. Using our NC preparation method, the encapsulation efficiency was higher than 88%, even at 10.0 mg/mL, which is a remarkable drug payload for this kind of system, corresponding to 0.2 mg of drug for each mg of nanocarrier wt./wt. (Table 2). Furthermore, the TMX loading yield was higher than 98%, at concentrations of 0.5 to 5.0 mg/mL and decreased at higher concentrations, indicating system saturation. This effect is clearly shown by the zeta potential data in the Figure 1c.

We evaluated the stability of the NC as a function of TMX concentration just after preparation and after 6 months of storage at 4 °C by measuring several parameters during storage, such as the zeta potential, mean size, polydispersity index, and TMX loading (Figure 1). No TMX precipitate was detected in NC containing 2.5 mg/mL and 5.0 mg/mL of TMX, which remained homogeneous and stable. NC containing 7.5 mg/mL and 10.0 mg/mL were unstable and the macroscopic aspect clearly indicated creaming and TMX crystal growth in the colloidal dispersion (data not shown). These changes in the stability of NC at TMX concentrations above 5.0 mg/mL occurred after 6 months of storage (Figure 1). The zeta potential of formulations changed (*p* < 0.05) with increasing TMX concentrations, showing that TMX exhibited effects at the NC surface. TMX-NC at 5.0 mg/mL showed excellent stability after 6-month storage period and was selected for further in vitro and in vivo experiments. This formulation presented a size smaller than 200 nm, a zeta potential of +42 mV and high payload.

**Figure 2:**
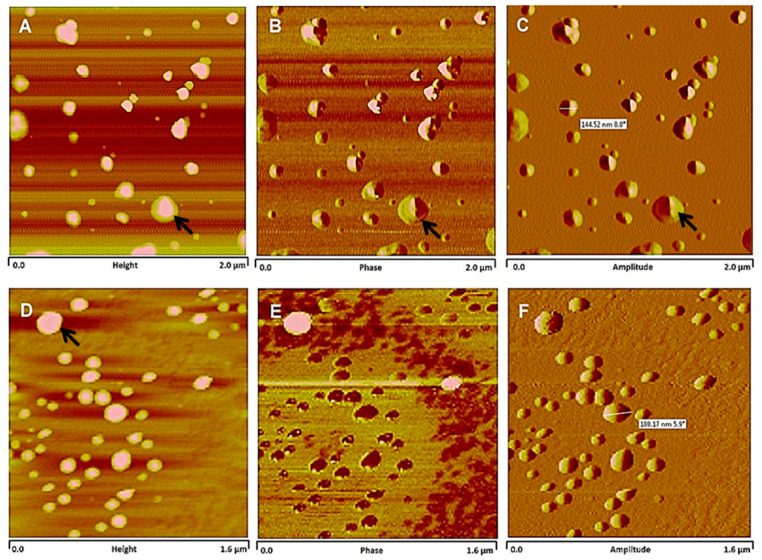
Atomic Force Microscopy images of blank-NC (**A**–**C**) and of TMX-NC at 5.0 mg/mL (**D**–**F**). Height (**A**,**D**); Phase (**B**,**E**); Amplitude (**C**,**F**) images are shown. Blank-NC images were 2.0 × 2.0 μm scan size and TMX-NC at 5.0 mg/mL images were 1.6 × 1.6 μm scan size. Arrows show core-shell nanostructures.

The geometric diameter and morphology of TMX-NC (5.0 mg/mL) and blank-NC were analyzed by Atomic Force Microscopy (AFM) in tapping mode and the images are shown in Figure 2. The values of *D*_h_ obtained by DLS are in agreement with the mean geometrical diameters obtained by AFM analyses (Table 2 and Figure 2). The AFM images evidenced nanostructures with a spherical shape in height, phase, and amplitude images (Figure 2). The size of TMX-loaded NC increased significantly (*p* < 0.05) compared to unloaded-NC, with an increased complexity of the structures observed by AFM. However, no aggregation or signs of TMX crystallization were detected by AFM analysis. In the heigth and amplitude images of the larger blank-NC (Figure 2A,C) we clearly observe core-shell type particles, in accordance with the vesicular nature of NC.

### 3.3. In Vitro Tamoxifen Solubility and Release

The experimental results of equilibrium solubility obtained for TMX in different simulated release media with surfactant (Polysorbate 80) are given in Table S1 (supplementary materials). Polysorbate 80 improved considerably TMX solubility In vitro, more than 100-fold in acidic medium and approximately 80-fold at pH 6.8 in intestinal fluid in agreement with the basic character of TMX. The 0.5% of Polysorbate 80 was the best surfactant concentration to improve solubility. It improved TMX solubility similarly after 24 h and 48 h at 37 °C. The solubility data for TMX reported in the literature is 1.02 µg/mL at 20 °C and 0.167 μg/mL at 25 °C [41,42]. TMX citrate aqueous solubility was reported to be 500 µg/mL at 37 °C [16]. Our data is sligthly different because of temperature and media.

**Figure 3:**
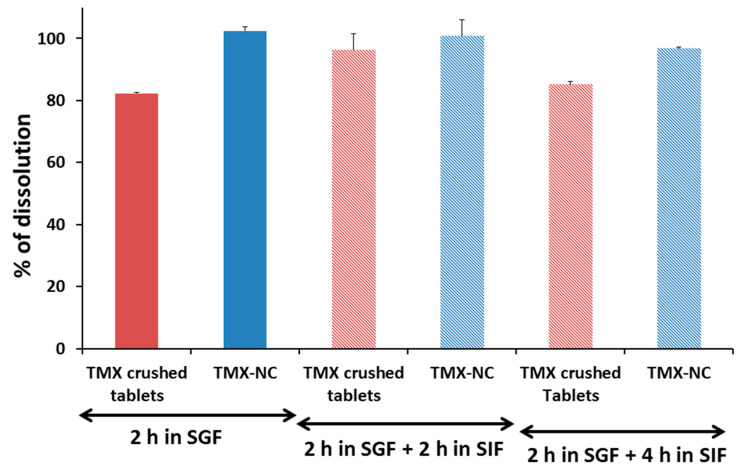
Tamoxifen dissolution percentage from crushed tablets and nanocapsules after 2 h incubation in simulated gastric fluid (SGF/Polysorbate 80 at 0.5%) at pH 1.2 followed by 2 h and 4 h in simulated intestinal fluid (SIF/Polysorbate 80 at 0.5%) at pH 6.8 using a dissolution apparatus without separation membrane. Data are mean and standard deviation of three independent experiments.

The percentages of dissolution of TMX after 2 h incubation in SGF media followed by more 2 h and 4 h in SIF is shown in Figure 3. The intrinsic dissolution of TMX shows a higher percentage in acidic media, in which TMX is more ionized. In the first 2 h in SGF, TMX dissolves to approximately 100% from NC (Figure 3). The TMX dissolution from crushed tablets and from NC was almost completed by using another type of release method without a separation membrane between a delivery system and the bulk medium. Both formulations release more than 80% of the TMX content in SGF with a very low additional release in SIF. As TMX is a basic drug, fast and high solubility is achieved in acidic SGF medium in 2 h.

### 3.4. Effect of Tamoxifen Nanocapsules on Tissue Parasite Burden

**Figure 4:**
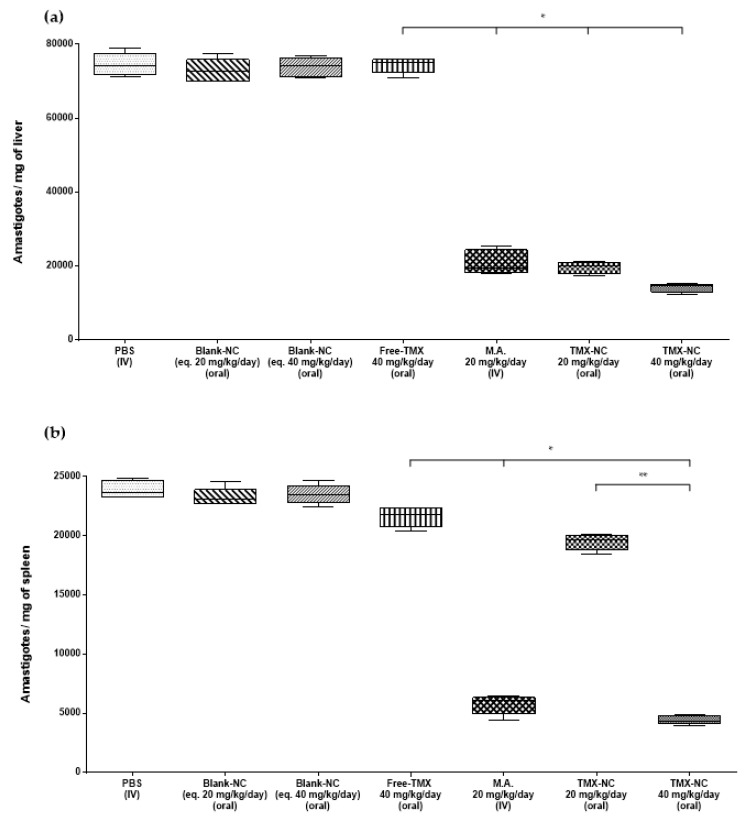
Parasite burden in liver (**a**) and spleen (**b**) of *L. infantum*-infected mice (*n* = 5 animals per group). The DNA parasite on tissues of the animals was quantified by qPCR. Box plots show the medians (horizontal lines across the box or points), interquartile ranges (vertical ends of the box or points), and whiskers (lines extending from the box or points to the highest and lowest values). The studies were performed in two independent experiments. Significant differences at *p* < 0.05 (*n* = 5) (Kruskal-Wallis followed by Dunn’s multiple comparison post-test) are indicated by bars and * between groups. * Indicates significant differences compared to the four first control groups ** Indicates significant difference compared to TMX-NC at 20 mg/kg/day group. No significant difference was observed among the four first control groups in the graphs. TMX: tamoxifen; M.A.: meglumine antimoniate.

**Figure 5:**
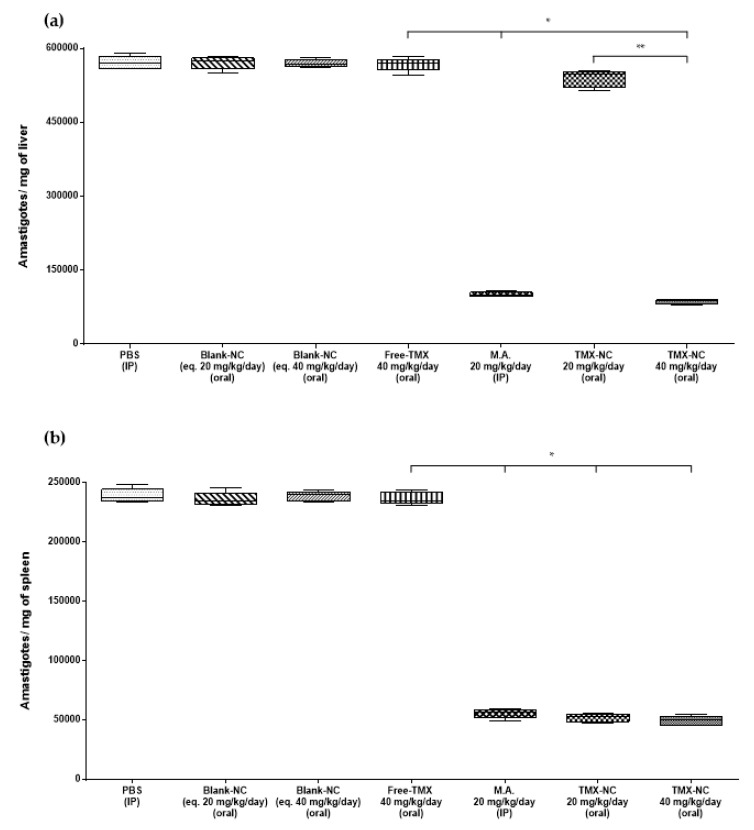
Parasite burden in the liver (**a**) and spleen (**b**) of *L. infantum*-infected hamsters (*n* = 5 animals per group). The parasite DNA in tissues of the animals was quantified by qPCR. Box plots show the medians (horizontal lines across the box or points), interquartile ranges (vertical ends of the box or points), and whiskers (lines extending from the box or points to the highest and lowest values). The studies were performed in two independent experiments. Significant differences at *p* < 0.05 (*n* = 5) (Kruskal-Wallis followed by Dunn’s multiple comparison post-test) are indicated by bars and stars between groups. * Indicates significant differences compared to the four first control groups ** Indicates significant difference compared to TMX-NC at 20 mg/kg/day group. No significant differences were observed among the four first control groups in the graphs. TMX: tamoxifen; M.A.: meglumine antimoniate.

In order to evaluate the efficacy of TMX-NC by the oral route, the parasite burden in the spleen and liver of mice and hamsters infected with *L. infantum* were evaluated 14 days after the end of treatment. Mice (Figure 4) and hamsters (Figure 5) treated orally with blank-NC equivalent to 20 mg/kg/day or 40 mg/kg/day and with free-TMX at 40 mg/kg/day by the oral route were unable to reduce the parasite burden in both organs of infected animals compared to the PBS group. Otherwise, the treatment with encapsulated drug (TMX-NC at 40 mg/kg/day) by the oral route had the same efficacy as parenteral meglumine antimoniate (standard treatment of VL) at 20 mg/kg/day. Both treatments induced a significant reduction of the parasite burden (*p* < 0.05) in liver and spleen of mice and hamsters, as shown in Figure 4 and Figure 5, respectively.

### 3.5. Biodistribution Analysis of Labeled Nanocapsules

**Figure 6:**
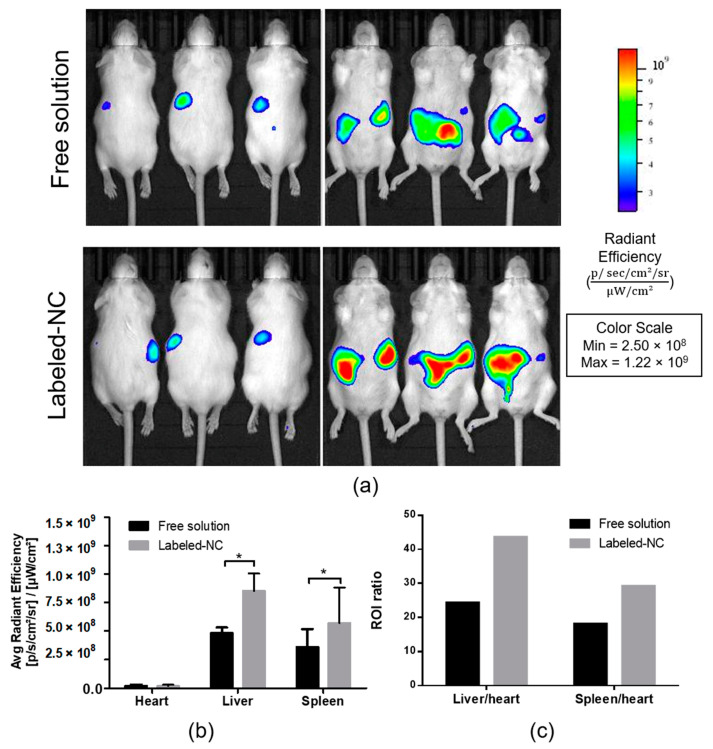
Biodistribution of labeled-NC and free solution of the dye after 30 min of oral administration. (**a**) In vivo fluorescence imaging in healthy mice (dorsal and ventral pictures); (**b**) Mean fluorescent intensity of dye solution in the heart, liver, and spleen in the control group and in labeled-NC groups of mice; (**c**) Regions of interest (ROI) calculated for levels of radiance in liver and spleen with respect to heart (non-interest). Data are expressed as mean ± SD (*n* = 3). * Indicates *p* < 0.001.

In vivo fluorescence imaging technique was used to investigate the biodistribution of labeled NC in mice. After 30 min of oral administration, an intense fluorescence signal was observed, concentrated predominantly in the liver and spleen (Figure 6a,b). The higher accumulation of NC in the liver and spleen was confirmed by the intensity of radiation and by the calculation of regions of interest (ROI) as shown in Figure 6b,c.

### 3.6. Histopathological Analysis

To evaluate the hepatic and splenic lesions correlated with *L. infantum* infection and the impact of different treatments, fragments of liver and spleen of mice and hamsters were collected and stained with H&E. These tissues were chosen because both of them are preferentially infected by *Leishmania infantum* and the parasites are responsible for many specific alterations in these tissues, caused in part by the response of immune system [9]. Thus, the treatment outcome in *Leishmania*-infected animal is clearly observed in both organs used in the standard experiments of chemotherapeutic efficacy [43].

**Figure 7:**
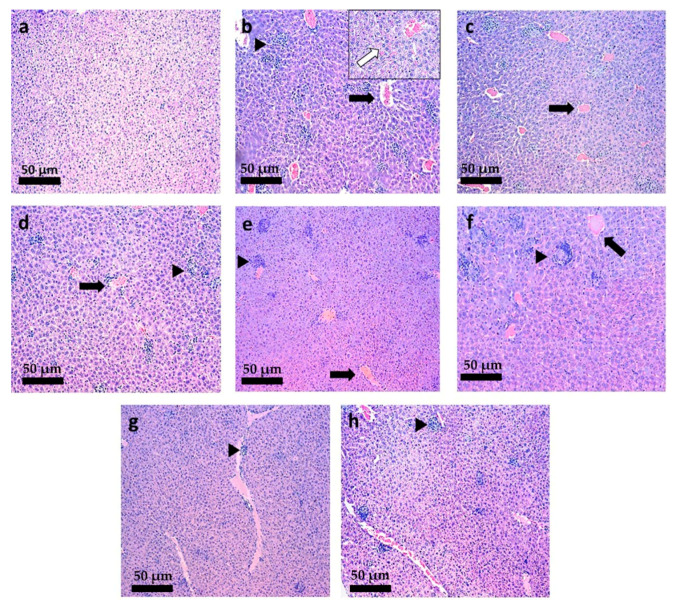
Photomicrographs of the liver of *L. infantum*-infected mice submitted to different treatments. (**a**) Non-infected mice and treated with PBS (IV); *L. infantum*-infected mice and treated with: (**b**) PBS by IV route; (**c**) Blank-NC equivalent to 20 mg/kg/day (oral); (**d**) Blank-NC equivalent to 40 mg/kg/day (oral); (**e**) Free-TMX at 40 mg/kg/day (oral); (**f**) meglumine antimoniate at 20 mg/kg/day (IV); (**g**) TMX-NC at 20 mg/kg/day (oral); (**h**) TMX-NC at 40 mg/kg/day (oral); *n* = 5 per group. Black arrow: hyperemia. Black arrowhead: granuloma. “Insert” in (**b**): white arrow showing hydropic degeneration and amastigote-filled granulomas. Hematoxillin-Eosin staining. 220 × magnification. (Bar = 50 µm).

In the liver of mice, histopathological changes such as hyperemia and the presence of small or large granulomas (qualitatively observed) were found in all groups infected by *L. infantum* (Figure 7b–h). Infected animals treated with intravenous PBS (Figure 7b) showed hydropic degeneration, amastigote-filled granulomas and marked hyperemia. The last lesion was also seen in groups treated with blank-NC (Figure 7c,d), free-TMX at 40 mg/kg/day by the oral route (Figure 7e) and with M.A. at 20 mg/kg/day by the IV route (Figure 7f). These lesions were not observed in mice treated with TMX-NC at 20 and 40 mg/kg/day (Figure 7g,h). Additionaly, granulomas were observed in animals infected by *L. infantum* in the liver of mice treated with TMX-NC at 20 and 40 mg/kg/day, but they were smaller and in smaller numbers (qualitatively observed) (Figure 7g,h).

**Figure 8:**
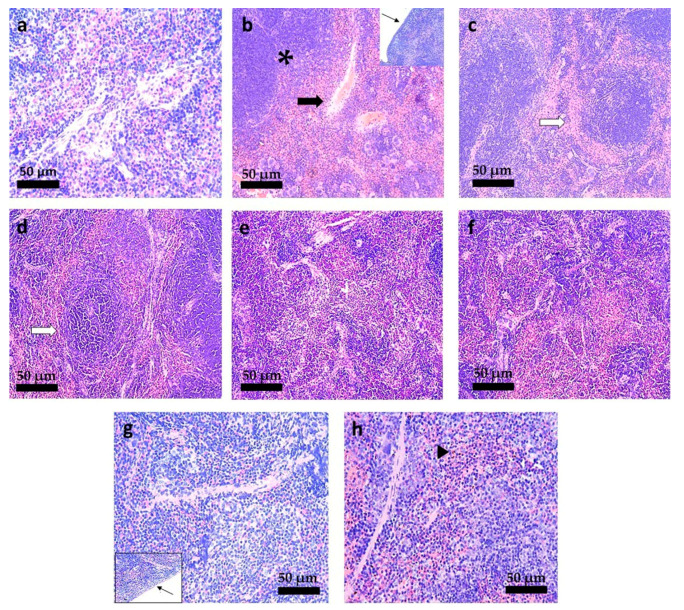
Photomicrographs of the spleen of *L. infantum*-infected mice submitted to different treatments (**a**) Non-infected mice and treated with PBS (IV); *L. infantum*-infected mice and treated with: (**b**) PBS by IV route; (**c**) Blank-NC equivalent to 20 mg/kg/day (oral); (**d**) Blank-NC equivalent to 40 mg/kg/day (oral); (**e**) Free-TMX at 40 mg/kg/day (oral); (**f**) meglumine antimoniate at 20 mg/kg/day (IV); (**g**) TMX-NC at 20 mg/kg/day (oral); (**h**) TMX-NC at 40 mg/kg/day (oral); *n* = 5 per group. Black arrow: hyperemia. Black arrowhead: granuloma. Black arrow: hyperemia. White arrow: enlarged lymphoid nodule. Asterisk: confluence of lymphoid nodules. Black arrowhead: hemosiderin pigments. “Inserts” in (**b**,**g**): fine black arrow showing capsule thickness. Hematoxillin-Eosin staining. 440 × magnification. (Bar = 50 µm).

The spleen of *L. infantum* infected-mice treated with intravenous PBS (Figure 8b) presented a histopathological picture characterized by marked hyperemia, confluence of lymphoid nodules, and increased capsule thickness (qualitatively observed). Mice treated with blank-NC by oral route showed an increase in lymphoid nodules (Figure 8c,d). On the other hand, the mice treated with TMX-NC at 20 and 40 mg/kg/day by the oral route presented a histopathological aspect compatible with normality (Figure 8g,h). The lesions, when found, were always focal with emphasis on the hemosiderin pigments (Figure 8h). The normal capsule thickness (qualitatively observed) and the reduction of other tissue changes induced by tissue amastigotes in the spleen of mice treated with TMX-NC at 20 and 40 mg/kg/day by the oral route (Figure 8g,h) suggest that TMX-loaded NC are able to reduce the parasitic burden and consequently to restore the architecture of the spleen.

**Figure 9:**
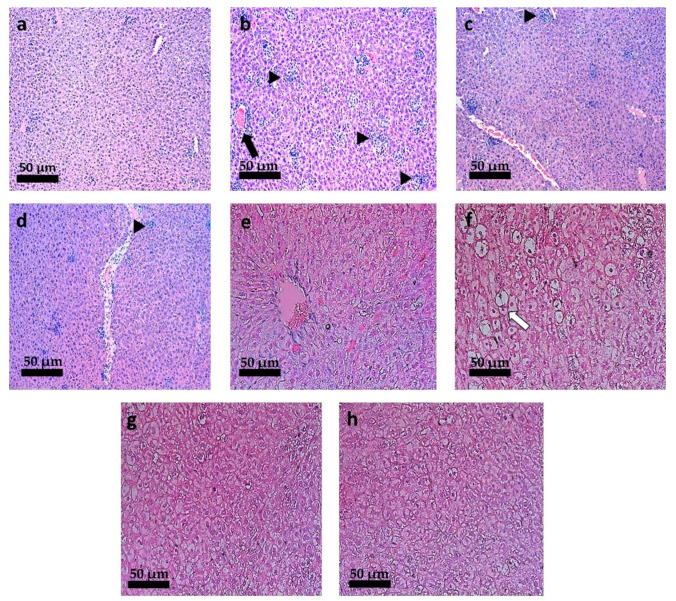
Photomicrographs of the liver of *L. infantum*-infected hamsters submitted to different treatments. (**a**) Non-infected hamsters and treated with PBS (IP); *L. infantum*-infected hamsters and treated with: (**b**) PBS by IP route; (**c**) Blank-NC equivalent to 20 mg/kg/day (oral); (**d**) Blank-NC equivalent to 40 mg/kg/day (oral); (**e**) Free-TMX at 40 mg/kg/day (oral); (**f**) meglumine antimoniate at 20 mg/kg/day (IP); (**g**) TMX-NC at 20 mg/kg/day (oral); (**h**) TMX-NC at 40 mg/kg/day (oral); *n* = 5 per group. Black arrow: hyperemia. Black arrowhead: granuloma. White arrow: hyperplasia and hypertrophy of the white pulp. Hematoxillin-Eosin staining. 220 × magnification. (Bar = 50 µm).

The histopathological changes in the liver of *L. infantum* infected-hamsters are shown in Figure 9. The hepatic architecture of *L. infantum* infected-hamsters treated with PBS (Figure 9b) showed hyperemia and numerous granuloma. The treatment with M.A. at 20 mg/kg/day by the oral route presented a histopathological picture characterized by marked hyperplasia and hypertrophy of the white pulp (Figure 9f). This lesion was not observed so markedly in any other animal group. Hamsters treated with TMX-NC at 20 and 40 mg/kg/day by the oral route presented a hepatic architecture compatible with normality (Figure 9g,h).

**Figure 10:**
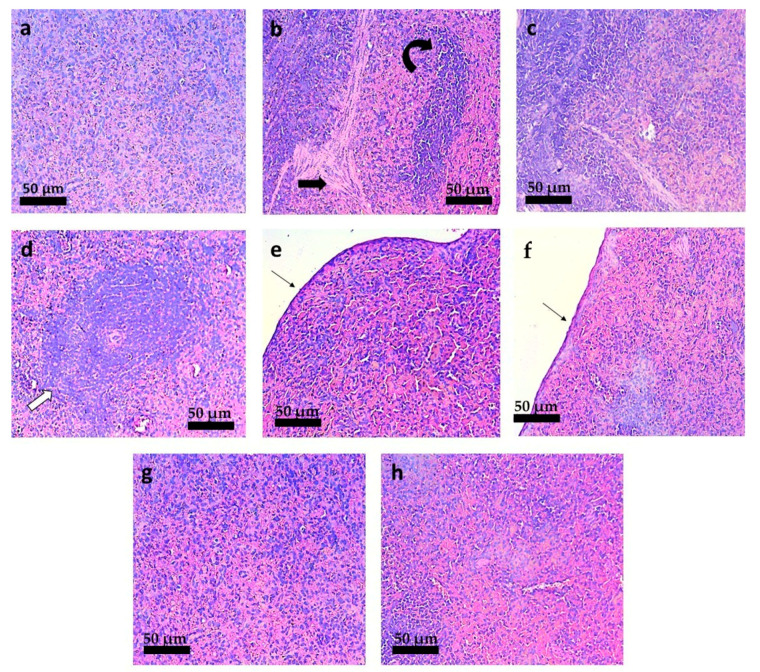
Photomicrographs of the spleen of *L. infantum*-infected hamsters submitted to different treatments. (**a**) Non-infected hamsters and treated with PBS (IP); *L. infantum*-infected hamsters and treated with: (**b**) PBS by IP route; (**c**) Blank-NC equivalent to20 mg/kg/day (oral); (**d**) Blank-NC equivalent to40 mg/kg/day (oral); (**e**) Free-TMX at 40 mg/kg/day (oral); (**f**) meglumine antimoniate at 20 mg/kg/day (IP); (**g**) TMX-NC at 20 mg/kg/day (oral); (**h**) TMX-NC at 40 mg/kg/day (oral); *n* = 5 per group. Black arrow: increase in trabeculae. Inclined black arrow: hyperplasia of the red pulp. White arrow: enlarged lymphoid nodule. Fine black arrow: capsule thickness. Hematoxillin-Eosin staining. 440 × magnification. (Bar = 50 µm).

The spleen of *L. infantum* infected-hamsters treated with intraperitoneal PBS show an increase in trabeculae and hyperplasia of the red pulp (Figure 10b). Unexpectedly, hamsters treated with blank-NC eq. to 40 mg/kg/day (Figure 10d) showed an increase (qualitatively observed) of the lymphoid nodules. These lesions were not observed in hamsters treated with TMX-NC at 20 and 40 mg/kg/day by the oral route, presenting a histopathological aspect compatible with normality (Figure 10f,g).

## 4. Discussion

The current VL treatment options involve parenteral administration for a prolonged time-schedule, which leads to several limitations, including poor patient compliance and the need for specialized medical facilities [6]. Failure to treat VL is often due to increased resistance of the parasite and to the toxicity of commonly used drugs [44]. Moreover, there are no effective vaccines to prevent leishmaniasis [45]. Therefore, in the past two decades, there has been a focus on developing new oral treatment for VL.

The strategy of repurposing drugs has been used as a fast alternative for different neglected diseases [4,23,46]. Previous data has shown a high TMX in vitro activity (IC_50_ 2.4 µM) against amastigotes and promastigotes of *Leishmania* and in vivo efficacy at doses of 20 mg/kg in infected-mice and hamsters [8]. Thus, the valuable, relatively fast and cost-effective strategy of repurposing TMX was used in this work, and an oral dosage form of polymeric NC was developed and characterized in order to be evaluated against VL experimental models.

Although TMX association to polymeric nanoparticles has been reported, low percentages of TMX encapsulation were observed. In the studies of Maji et al., 2014 [47], the TMX % loading in NC of poly(lactic-*co*-glycolic acid) (PLGA) and polyvinyl alcohol was only 27.16%. In the studies of Yu et al., 2020 [48], the percentage of TMX in nanoparticles of PLGA-PEG-PLGA was 49%. Other authors obtained maximum encapsulation of 64% for TMX in nanoparticles prepared with poly-ε-caprolactone using (1:20) ratio of drug/polymer and poloxamer 188 as stabilizer [49]. However, in our study with the diblock copolymer PLA_22k_-*b*-PEG_5k_ used to prepare NC formulations at 0.5 to 5.0 mg/mL of TMX, entrapment yields higher than 98% were achieved, most likely due to the affinity of TMX for the medium chain triglyceride core of the NC. As mentioned previously, TMX is a lipophilic base with a log *P* value of 7.1 and a pKa value of 8.7 [15,16], which explains the high payload in oil-filled polymeric NC. Thus, the use of TMX free base improved the encapsulation efficiency compared with previous studies [47,48,49,50], because TMX base partition coefficient favored association to the NC oily core.

**Figure 11:**
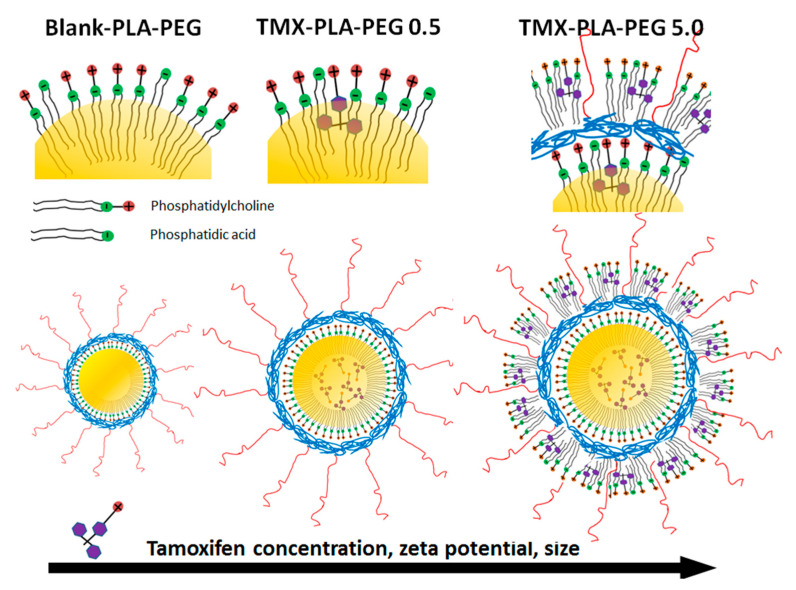
Schematic representation of hypothetic PEG-PLA NC structural organization based on the physicochemical features and effects of TMX on size and surface charge.

The PEG-PLA diblock copolymer can be synthesized to tune the hydrophilic hydrophobic balance of the polymer. Depending on the length of the hydrophobic PLA block and total molar mass of PEG-PLA, micelles to hard nanospheres can be obtained [51]. In order to form a polymeric shell capable of stabilizing the oily core of polymeric NC, a sufficiently long PLA block is necessary, such as in the case of PLA_22k_-*b*-PEG_5k_ with the (4:1) PLA/PEG ratio, allowing the formation of core-shell NC. Although PEG_5k_ chains are expected to shield surface charges, TMX has a large influence on the NC surface charges, indicating ionization of the drug and strong interaction with surfactants at the NC surface. The high positive NC zeta potentials contribute to the colloidal stability of the dispersion and to the interaction with cell membranes. Strong interaction of phospholipids has been described for TMX and it may be related to the effect on NC surface charge, and most likely involved in the stabilization of the oil core [51]. Zeta potential measurements indicated that part of the TMX was associated with the NC surface, most likely interacting also with impurities of lecithin (Lipoid^®^S75), such as phosphatidic acid, as previously discussed [52,53]. This strong effect of TMX on NC surface charge is related to ionization of compounds near the NC interface with water. Similar results were found and discussed previously with halofantrine base in NC [54]. The study reported increasing sizes and zeta potential with increasing drug concentration of this antimalarial drug, with a molecular structure similar to TMX. A hyphotetic scheme of the structural organization of TMX-loaded in PEG-PLA NC is proposed in Figure 11. The size of the NC is an important parameter that influences drug biodistribution in the body [27]. The effect of TMX on sizes may be explained by the association with the oily core and, at higher concentrations, also with the surface charged lipids, as proposed in Figure 11. The NC prepared in the present study have sizes below 200 nm with narrow size distribution, TMX encapsulated at high payloads with good physical stability over 6 months (2.5–5 mg/mL), and no drug precipitation, which are all promising features to apply these devices in VL treatment.

When we studied dissolution of both formulations dispersed directly in bulk release media, a complete release was observed in SGF. A similar behaviour was previously observed and discussed with halofantrine in NC [54]. The drug release process is equilibrium, and it is shown in Figure 3 that more than 80% of TMX from crushed tablets and around 100% from NC was released in vitro after 2 h. In fact, TMX forms a soluble salt at a pH of 1.2 in SGF. TMX is normally absorbed in TGI with 100% bioavailability in humans, with fast intestinal and hepatic metabolism. Fast release from NC is expected, because nanoparticles provide a higher interfacial area (190 nm particles) for TMX dissolution compared with coarse crushed tablets suspension in our experimental condition. Thus, TMX partition in favor of acidic media was accelerated by high interfacial area of NC. Previously, we have shown that NC is a permeable nanocarrier that can release rapidly a drug by partition [54]. The polymer membrane of NC is very thin and does not present a major barrier to drug release. In the case of NC, drug release depends on partition with the external medium, and in particular with drug acceptors in the bulk media [54]. The effects of drug solubility on the external medium are a more important factor. In a previous study, a tamoxifen-phospholipid complex exhibited increased solubility, dissolution rate, and considerable enhancement in the oral bioavailability in comparison to free-tamoxifen [55]. This enhancement was attributed to the improvement of the aqueous solubility of the complex and a probable decrease in its extent of intestinal and hepatic metabolism.

Attili-Qadri et al., 2013 [26] demonstrated elegantly that NC was rapidly absorbed from TGI and provides a modification of biodistribution and release rate in the blood. These authors also demonstrated the theory of intestinal absorption of this nanocarrier in its intact form. Herein, the biodistribution study conducted 30 min after administration showed clearly that NC accumulates at a higher extent in the liver and spleen of mice. Thus, it is most likely that NC is rapidly absorbed, and the TMX fraction associated with the carrier was able to accumulate in the mononuclear phagocyte system (MPS) inducing the best efficacy effect observed in *L. infantum*-infected mice, compared with free-TMX. This difference in biodistribution indicates that NC seems not to be degraded in the first 30 min after administration. In vivo accumulation of the NC in the liver and spleen after 30 min post-administration seems to improve the delivery of TMX to these infected-organs by the oral route. In this sense, biodistribution data from dye labeled-NC clearly show that, after oral administration, the fluorescence due to NC administration accumulates in the liver and spleen to a higher extent compared to the dye in solution. This corroborates with the intestinal absorption of this nanocarrier [26] and reinforces the hypothesis of the lymphatic absorption pathway and accumulation in MPS in the liver and in spleen. Both organs have high blood flow and, to reduce a possible influence of blood flow in NC accumulation, the ROI ratio was calculated comparing radiance ratios of liver/heart and spleen/heart, because the heart has a similarly high blood flow. Encapsulation increases targeting to the liver and spleen by two-fold, where a ROI ratio higher than 1.5 already indicates selective targeting to an organ. The ROI, in this sense, is indicative of passive targeting of drug loaded NC to the liver and spleen by oral administration. Furthermore, the NCs are actively phagocytosed by macrophages [53], representing an example of passive targeting to MPS and an outstanding advantage in the treatment of VL.

The effects of surface chemistry and dose of PLA nanocapsules on biodistribution and clearance were previously reported [52,53,56]. The nanocapsules are composed of phospholipids (mainly phosphatidylcholine) and medium-chain triglycerides, fully biocompatible, innocuous, and safe. The nanocapsule polymeric wall is a polyester (polylactide polymer), which is biodegradable by esterases in lactic acid. Lactic acid enters the metabolic pathway, where it is further degraded into CO_2_ and water. The covalently linked PEG chain length is short (5000 Da) and is eliminated by glomerular filtration in the kidneys.

Considering that there is no LV chemotherapy available by oral route in Brazil and considering the global resistance to oral miltefosine in various parts of the world, the present study investigated the effect of oral TMX-NC on parasite burden and on the histopathological characteristics in *L. infantum*-infected mice and hamsters. Indicated for treatment or prevention of human breast cancer, TMX is used continuously for 5–10 years with daily doses of 10–40 mg/day and shows a good safety profile [57]. In the literature, doses administered for the experimental treatment of breast tumors in mice range from 45 to 400 mg/kg/day [58,59]. In TMX repositioning for the treatment of leishmaniasis, doses of 6.5–26 mg/kg/day are effective in mice [20,60]. Doses in mice are much higher than the daily doses given to humans, most likely due to the different metabolism of the two species. During our experiments, no clinical toxicity was observed in the animals even at the highest dose of 40 mg/kg/day. Our histopathological analysis of the liver and spleen showed that our formulation was able to reduce alterations in these organs. Therefore, the highest doses tested orally in our experimental models of visceral leishmaniasis in mice and hamsters, when transposed to human doses, will be within the range already studied clinically, which was already found not to induce significant adverse effects in patients [57].

We demonstrate the efficacy of the TMX-NC at 40 mg/kg/day in reduction of parasite burden in the liver and spleen of both experimental models. Additionally, oral TMX-NC at 20 and 40 mg/kg/day visually induced a decrease in the number and size of granuloma in the liver of mice. Moreover, this treatment was able to reduce the parasitic burden and consequently restore the architecture of the spleen of mice and hamsters. Likewise, Branquinho et al., 2020 [32], showed a high level of oral efficacy of LYC loaded in NC during the acute and chronic phases of *T. cruzi* infection and dramatically reduced parasite burden in heart tissue. Furthermore, the LYC-NC reduced heart pathological damages in mice infected with *T. cruzi* strain resistant to classical drugs [32]. The last study and our present work indicate that NC formulations are able to efficiently deliver a drug at certain target-organs even when administered by the oral route. The *L. infantum* strain used in our study (OP46) was previously characterized in terms of its tissue dynamics after experimental infection in hamsters (*M. auratus*), that reproduce several typical aspects of human VL [38]. The authors demonstrated that the OP46 strain has high infectivity compared to a reference strain of *L. infantum* (WHO/MHOM/BR/74/PP75) and after the 6th week of infection, the percentage of positive parasite DNA and the parasite burden in the liver were higher in comparison to splenic tissue [38]. These data are in agreement with ours results of tissue parasite burden in the liver and spleen of animals. Furthermore, the high infectivity of the OP46 strain described in hamsters [38,61] can explain the lower reduction of the parasite burden in the liver of hamsters treated with TMX-NC at 20 mg/kg/day. Similarly to our work, the high reactivity of lymphoid nodules in the white pulp was also demonstrated by Reis et al. 2017 [37], who suggested it to be a lesion related to the high parasite burden of *L. infantum* (strain OP46) in the spleen of mice.

The preferential accumulation of the NC in the liver and spleen evidenced through biodistribution studies partially explains the reduction in parasite burden in both organs compared to the free-TMX. Nanoencapsulation can increase the therapeutic efficacy of drugs because their biodistribution follows that of the carrier, rather than depend on the physicochemical properties of the active molecule itself [62].

Finally, for the first time, formulations of TMX-loaded in NC were developed and evaluated against VL in two animal models. The results indicate a promising strategy of repurposing TMX as an oral dosage form loaded in polymeric NC. It also showed better efficacy/histophatological profile compared to classical treatment with M.A. This formulation will be investigated in further pre-clinical studies. Altogether, the results obtained in this work confirmed that TMX-NC is an advantageous antileishmanial formulation for experimental treatment of *L. infantum*.

## 5. Conclusions

The overall physicochemical characterization demonstrated that PLA-PEG NC are a promising option for oral delivery of TMX. Polymeric NC containing TMX can be prepared at high payloads from preformed polymer using a simple method with a prolonged TMX release profile. The formulations were stable under storage in liquid form and this can be further improved by freeze-drying (studies are in progress). Our data show that TMX-NC at 40 mg/kg/day administered by the oral route was able to significantly reduce the parasite burden in the liver and spleen of mice and hamsters infected with *L. infantum* (OP46 strain). Pathological damages induced by the infection were also reduced and clearly evidenced the NC accumulation in the liver and spleen, which explains in part the better efficacy profile of TMX-NC against *Leishmania*. Thus, TMX repurposing in NC in experimental VL is an effective strategy and particularly attractive to provide currently unavailable oral treatment for human VL, which merits further investigations.

## Figures and Tables

**Table 1:** Effect of different formulation variables on the hydrodynamic diameter and polydispersity index of tamoxifen-loaded nanocapsules.

Formulation	TMX (mg/mL)	Ethanol/Acetone Ratio (*v*/*v*)	Oil Phase (mL)	Aqueous Phase (mL)	Oil Phase/Aqueous Phase Ratio (*v*/*v*)	*Z*-Average *D*_h_ ± SD	PdI ± SD
F1	0.0	1:1	2	4	1:2	174.5 ± 11	0.157 ± 0.09
F2	2.5	1:1	2	4	1:2	215.4 ± 13	0.231 ± 0.10
F3	5.0	1:1	2	4	1:2	235.5 ± 9	0.262 ± 0.09
F4	0.0	2:2	4	8	1:2	136.9 ± 7	0.145 ± 0.05
F5	2.5	2:2	4	8	1:2	185.7 ± 4	0.118 ± 0.08
F6	5.0	2:2	4	8	1:2	191.5 ± 5	0.131 ± 0.09
F7	0.0	3:1	4	8	1:2	154.7 ± 8	0.156 ± 0.05
F8	2.5	3:1	4	8	1:2	203.1 ± 11	0.242 ± 0.06
F9	5.0	3:1	4	8	1:2	215.1 ± 12	0.261 ± 0.12
F10	0.0	3:1	4	16	1:4	121.7 ± 7	0.171 ± 0.08
F11	2.5	3:1	4	16	1:4	356.6 ± 14 ^1^	0.392 ± 0.09 ^1^
F12	5.0	3:1	4	16	1:4	405.8 ± 12 ^2^	0.42 ± 0.13 ^2^

^1^ Indicates significant differences compared to F5 (*p* < 0.05). ^2^ Indicates significant differences compared to F6 (*p* < 0.05). The final volume of all colloidal dispersion was 2 mL after solvent evaporation and concentration. TMX: tamoxifen; PdI: polydispersity index; *D*_h_: hydrodynamic diameter; SD: standard deviation (*n* = 3) on measurements of three NC batches.

**Table 2:** Physicochemical characterization of tamoxifen-loaded PEG-PLA nanocapsules.

TMX (mg/mL)	*Z*-Average *D*_h_ ± SD	PdI ± SD	Geometric Diameter ^a^ (nm) (AFM)	pH ± SD	Loading Yield (%) ± SD	Encapsulation Efficiency (%) ± SD	TMX Payload (µg/mg)
0.0	136.9 ± 10	0.157 ± 0.07	136.5 ± 7.5	6.10 ± 0.05	-	-	-
0.5	152.9 ± 11	0.163 ± 0.05	ND	5.82 ± 0.05	98.19 ± 0.07	96.48 ± 0.05	11,11
1.5	163.9 ± 8.5 ^1^	0.198 ± 0.03	ND	5.53 ± 0.08	98.54 ± 0.07	96.24 ± 0.07	32.61
2.5	185.7 ± 6 ^1^	0.103 ± 0.05	ND	5.14 ± 0.03 ^1^	98.85 ± 0.09	96.84 ± 0.06	53.19
5.0	191.5 ± 7 ^1^	0.128 ± 0.06	180.17 ± 5.7 ^1^	4.77 ± 0.02 ^1^	98.68 ± 0.11	93.90 ± 0.14	101.01
7.5	255.8 ± 9 ^1^	0.261 ± 0.04 ^1^	ND	4.25 ± 0.06 ^1^	95.21 ± 0.18	90.11 ± 0.19	144.23
10.0	306.2 ± 9.5 ^1^	0.374 ± 0.8 ^1^	ND	3.82 ± 0.09 ^1^	93.54 ± 0.09	88.45 ± 0.17 ^1^	183.49

^1^ Indicates significant differences compared to blank-NC (*p* < 0.05). ^a^ Geometric diameters were measured from topographical profiles of AFM considering the width of the spheres at half height reported for 60 particles. Payload is the drug mass carried by a specific mass of the nanocarrier and it is calculated as the mass of TMX encapsulated (µg) divided by the total mass of NC excipients (µg) for each ml of formulation. TMX: tamoxifen; PdI: polydispersity index; D*_h_*: hydrodynamic diameter; SD: standard deviation (*n* = 3) of measurements of three NC batches; ND: not determined.

## Data Availability

The data presented in this study are available in the research article and supplementary material here.

## Supplementary Materials

The following are available online at https://www.mdpi.com/article/10.3390/pharmaceutics13071061/s1, Table S1: Equilibrium solubility (µg/mL) determined for tamoxifen base in simulated gastric fluid and simulated intestinal fluid with Polysorbate 80 at 0.25–0.5%.
