# Supplementary material for: Repositioning of Tamoxifen in Surface-Modified Nanocapsules as a Promising Oral Treatment for Visceral Leishmaniasis
Source: Pharmaceutics. 2021 Jul 10;13(7):1061. doi: 10.3390/pharmaceutics13071061 (PMC8309129; doi:10.3390/pharmaceutics13071061)
Supplement: Supplementary file 1 [file pharmaceutics-13-01061-s001.zip › pharmaceutics-1250425-supplementary.pdf]

pharmaceutics

Supplementary Materials: Repositioning of Tamoxifen in Surface-Modified Nanocapsules as a Promising Oral Treatment for Visceral Leishmaniasis

Débora Faria Silva, Levi Eduardo Soares Reis, Marina Guimarães Carvalho Machado, Douglas Daniel Dophine, Vinicius Roberto de Andrade, Wanderson Geraldo de Lima, Margareth Spangler Andrade, José Mário Carneiro Vilela, Alexandre Barbosa Reis, Gwenaelle Pound-Lana, Simone Aparecida Rezende and Vanessa Carla Furtado Mosqueira

1. Preparation of TMX base from TMX citrate

Briefly, 10 g of TMX citrate was dissolved in 20 mL of methanol and the solution transferred to a separation funnel containing 100 mL of ultrapure water supersaturated with sodium bicarbonate and vigorously mixed. Then the TMX free base was extracted with three portions of chloroform (30 mL) under vigorous shaking. The organic phases were combined and washed 3 times with ultrapure water. The chloroform phase was separated and dried over anhydrous magnesium sulphate and filtered. The solvent was evaporated to dryness under reduced pressure in a rotary evaporator (Heidolph, Germany). The gravimetric yield of TMX free base extraction was 90.3%. The purity of TMX free base was verified by melting point analysis in a heated capillary-type instrument Büchi B-540 (Büchi, Switzerland) and compared to the literature value of 97 °C [1]. Furthermore, under HPLC analysis of TMX with diode array detection no secondary peak was observed.

2. Chromatographic conditions for TMX quantification

The chromatographic method for TMX quantification in TMX-NC formulations was developed from the method described previously [2] with some modifications in parameters including the detection wavelength, flow and volume of injection. The HPLC-UV/Vis system consisted of a Waters Alliance E2695 separation module, autosampler, pump, column oven and a Waters 2489 UV detector set at 254 nm (Waters Corporation, Milford, MA, USA). The separation was performed using a 150 mm × 4.6 mm C18 Gemini-NX Phenomenex® column with a 5 µm particle size and protected by a Gemini-NX C18 Phenomenex® security guard pre-column (2 mm × 4.6 mm, 3 µm). The mobile phase composed of methanol: aqueous ammonium acetate 1.5% p/v (85:15 *v*/*v*) was prepared daily, filtered through a 0.45 µm membrane and degassed in an ultrasonic bath before use. Chromatographic separation was carried out at a 1.0 mL/min flow rate with isocratic elution at 40 °C column temperature with an injection volume of 60 µL and run time of 10 min. The retention time of TMX was 5.25 min. The method was selective to quantify TMX in the presence of the NC excipients and no interference with the TMX peak was observed. The method was linear in the working range of 0.5–25 µg/mL (coefficient of regression, r^2 = 0.998). The limit of detection (LOD) and limit of quantification (LOQ) were 0.51 and 1.55 µg/ml, according to current pharmaceutical guidelines of the International Conference of Harmonization (ICH) [3].

3. In vitro tamoxifen solubility study

Tween®80 at 0.5% was added to the media to improve TMX solubility. An excess of free-TMX (10 mg) was added to 10 ml of each medium and they were maintained under magnetic stirring (300 rpm) for 24 and 48 hours at 37° C in triplicate. Then, samples (500 µL) (*n*=3) were collected from the external medium and mixed with 500 µL of acetonitrile under vortex mixing for 15 min (Vortex Instrument, IKA, Germany) and centrifuged at 500 × *g* for 15 min (Centrifuge 5415 D, Eppendorf, Hamburg, Germany). The supernatant

was collected, filtered (0.45 μm filter, Millipore®) and assayed by HPLC-UV according to the chromatography conditions described above.

Table S1. Equilibrium solubility (μg/ml) determined for tamoxifen in simulated gastric fluid and simulated intestinal fluid with Polysorbate 80 at 0.25–0.5%.

Release Medium (37 °C)	Tamoxifen Solubility (μg/mL) Mean ± SD	
	24 h	48 h
PBS pH 7.4	0.91 ± 0.08	0.98 ± 0.07
SGF + Polysorbate 80 0.25%	62.35 ± 0.07	64.5 ± 0.04
SGF + Polysorbate 80 0.5%	98.75 ± 1.05 [1]	104.5 ± 0.03 [1]
SIF + Polysorbate 80 0.25%	45.70 ± 1.04	46.32 ± 0.02
SIF + Polysorbate 80 0.5%	76.50 ± 1.09 [2]	80.4 ± 0.03 [2]

[1]Significant difference compared to SGF/ Polysorbate 80 0.25% ($p < 0.05$). [2]Significant difference compared to SIF/ Polysorbate 80 0.25% ($p < 0.05$); PBS: phosphate buffered saline; SGF: simulated gastric fluid (pH 1.2); SIF: simulated intestinal fluid (pH 6.8); SD: standard deviation.

References

1. Santana, D.P.; Braga, R.M.C.; Strattmman, R.; Albuquerque, M.M.; Bedor, D.C.G.; Leal, L.B.; Silva, J.A. Reversed phase HPLC determination of tamoxifen in dog plasma and its pharmaco-kinetics after a single oral dose administration. *Quím. Nova* **2008**, *31*, 47–52, doi:10.1590/S0100-40422008000100010.
2. Jena, S.K.; Sangamwar, A.T. Polymeric micelles of amphiphilic graft copolymer of α-tocopherol succinate-g-carboxymethyl chitosan for tamoxifen delivery: synthesis, characterization and in vivo pharmacokinetic study. *Carbohyd. Polym.* **2016**, *151*, 1162-1174, doi: 10.1016/j.carbpol.2016.06.078.
3. Validation of analytical procedures: text and methodology Q2 (R1). Proceedings of the International conference on harmonization, Geneva, Switzerland, **2005**. Available online: https://www.ema.europa.eu/en/ich-q2-r1-validation-analytical-procedures-text-methodology#current-effective-version-section (acessed on 04 04 2021).
